# The Research Progress of Vision-Based Artificial Intelligence in Smart Pig Farming

**DOI:** 10.3390/s22176541

**Published:** 2022-08-30

**Authors:** Shunli Wang, Honghua Jiang, Yongliang Qiao, Shuzhen Jiang, Huaiqin Lin, Qian Sun

**Affiliations:** 1 College of Information Science and Engineering, Shandong Agricultural University, Tai’an 271018, China; 2 Australian Centre for Field Robotics (ACFR), Faculty of Engineering, The University of Sydney, Sydney, NSW 2006, Australia; 3 College of Animal Science and Veterinary Medicine, Shandong Agricultural University, Tai’an 271018, China

**Keywords:** precision pig farming, livestock farming, artificial intelligence, pig detection and tracking, behavior recognition, sound recognition

## Abstract

Pork accounts for an important proportion of livestock products. For pig farming, a lot of manpower, material resources and time are required to monitor pig health and welfare. As the number of pigs in farming increases, the continued use of traditional monitoring methods may cause stress and harm to pigs and farmers and affect pig health and welfare as well as farming economic output. In addition, the application of artificial intelligence has become a core part of smart pig farming. The precision pig farming system uses sensors such as cameras and radio frequency identification to monitor biometric information such as pig sound and pig behavior in real-time and convert them into key indicators of pig health and welfare. By analyzing the key indicators, problems in pig health and welfare can be detected early, and timely intervention and treatment can be provided, which helps to improve the production and economic efficiency of pig farming. This paper studies more than 150 papers on precision pig farming and summarizes and evaluates the application of artificial intelligence technologies to pig detection, tracking, behavior recognition and sound recognition. Finally, we summarize and discuss the opportunities and challenges of precision pig farming.

## 1. Introduction

As the world’s population continues to increase, the global demand for sustainable animal products is increasing [1]. According to the latest data from the “China Statistical Yearbook-2021” [2], China’s large livestock population is 102.651 million, and the total output of pork is 41.133 million tons, which is the largest proportion of all meat, accounting for about 53.00% of the total meat. Moreover, pork accounts for a large proportion of livestock products in many countries [3]. Therefore, the impact of pork as a sustainable livestock product is critical to global food security.

While the global economy grows and people become more aware of healthy food, there is a greater concern for pig health and welfare [4,5]. Specifically, the influence of COVID-19 in the last two years has made animal health, welfare, livestock product quality and smart livestock farming become a central topic among consumers and farmers [6,7]. In Europe, with the development of market strategies, animal welfare standards have been continuously improved, and minimum welfare standards have been formulated in the process of pig farming, transportation and slaughtering [8,9,10]. Animal welfare can currently be assessed in three areas: natural life, emotional state and basic health and function [11]. In terms of pig health and welfare, it can be assessed from some factors such as appearance phenotype (e.g., body size, weight), behavioral performance or sound recognition [12]. In tradition, pig welfare and health were judged by farmers based on personal experience observations [13]. However, human observation can not satisfy the requirements of real-time monitoring in pig farming [14]. To improve the efficiency and sustainability of pig farming, new technologies such as smart sensors, internet of things (IoT) and artificial intelligence (AI) have been gradually used in the pig industry [15,16].

Precision livestock farming (PLF) technology enables the management of livestock herds using technologies such as AI and IoT [17,18,19,20]. The PLF combines sensors and devices with intelligent software to extract key farming information and then provides management strategies that enable famers to monitor animals automatically to improve animal health, welfare, yields and environmental impacts. In the framework of PLF, sensors (e.g., microphones, cameras) are used to monitor the animal appearance prototypes and then utilize engineering techniques to automatically recognize animal behavior or growth situation for the final decision-making of livestock farming [21]. In the modern smart livestock industry, PLF is becoming increasingly important because it is difficult to achieve intensive agriculture and single animal care without the help of technology [22,23]. AI has increased the usability of sensors and electronic devices in the smart livestock industry, which significantly facilitates the development of pig and other domesticated animals’ farming.

Driven by information technology, the pig farming model is undergoing an unprecedented great change. The combination of AI and pig farming can realize intelligent perception, accurately understand the pig behavior (e.g., estrus, feeding, walking and standing), accurately monitor physiological conditions of pigs through their sound signals and carry out fine and personalized feeding management, to make the pig farming process more scientific, intelligent and modern [24].

This paper summarizes the current research progress of AI in pig farming, which includes the application of AI systems in pig farming, AI-based pig detection and tracking, AI-based pig behavior recognition and pig sound recognition. Then we summarize and discuss the challenges and limitations. Finally, this paper expounds on the opportunities of smart pig farming.

## 2. The Framework of Precision Pig Farming

**Figure 1:**
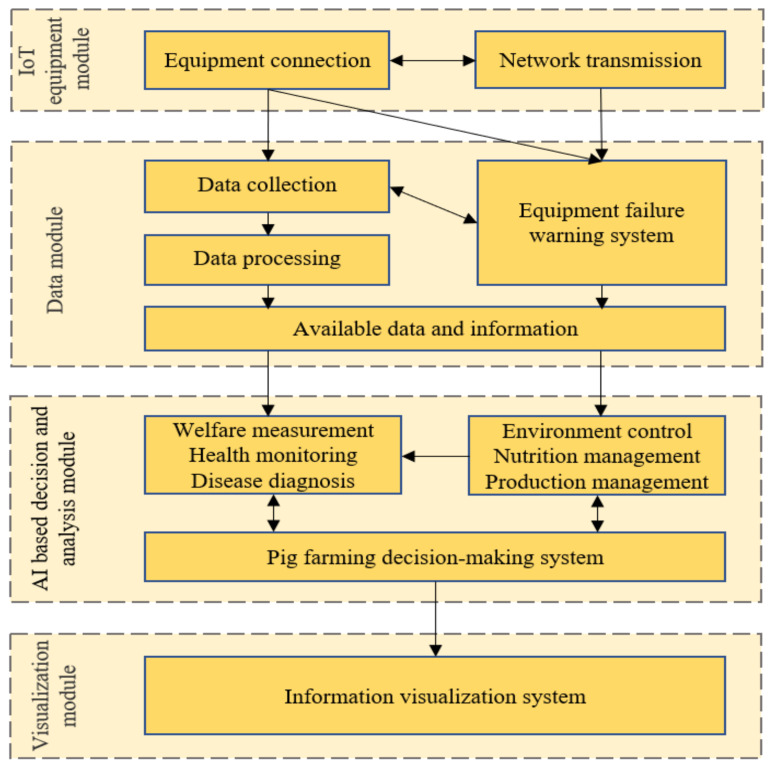
Precision pig farming framework.

Precision pig farming aims to improve the ability of farmers to manage large pig herds and enhance the effective monitoring and management of each pig’s health and welfare [17,22,25,26,27]. A typical precision pig farming system is composed of four key modules (Figure 1): IoT equipment module, data module, AI-based decision and analysis module and visualization module [11,27]. In recent years, with the development of big data, AI and IoT, precision pig farming has made significant developments [28].

- The IoT equipment module contains data collection sensors, environment monitoring and control devices, connection and network transmission devices and other related facilities. The IoT system should be operated properly in a harsh environment to ensure data and information can meet pig farming’s needs [29].
- The data module consists of data collection, data processing, data storage and an equipment failure warning system [30]. The data collection and processing are mainly responsible for processing data collected by IoT equipment, which generates usable data and information. The equipment failure warning system mainly monitors sensors, automatic feeders, pumps and other physical equipment to collect data normally.
- The AI-based decision and analysis module contains pig health and welfare evaluation, disease diagnosis, environment control, nutrition and production management and a pig farm decision-making system. The pig’s welfare is usually expressed by the pig’s behavior [31], and the pig’s health, welfare and disease diagnosis can be evaluated through a decision making-system using vision and sound signals. On the other hand, an AI-based decision-making system controls the operation of physical equipment involving environmental control and nutrition management to increase pig health and welfare, mainly based on the results of the above analysis [28,32].
- The visualization module provides farmers with visual information and displays valid information output from other modules.

**Figure 2:**
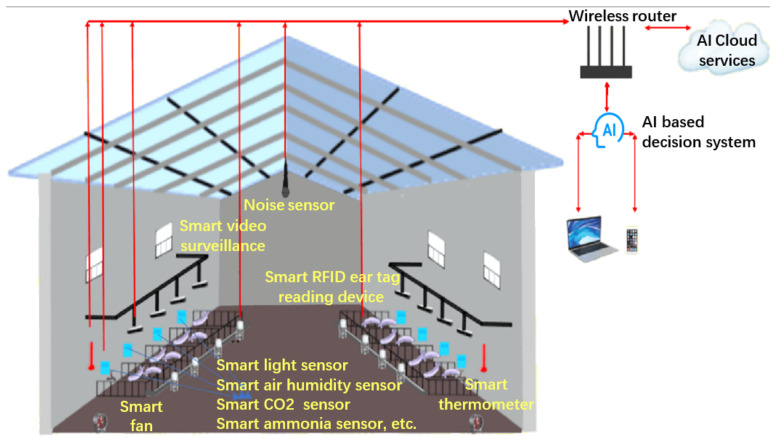
Implementation of precision pig farming technology.

Precision pig farming uses technologies such as IoT and AI to continuously monitor pig health and welfare with the following main functions [33]: (1) The IoT is mainly used in the design and layout of temperature, humidity and other sensors, as well as associated networking equipment and data collection. The industrial Internet is mainly used to transmit the data to the server; AI and cloud computing are mainly applied to feature extraction, data analysis, modeling and decision-making [34,35,36]. Here reliability is one of the keys to the early success of the deployment of precision pig systems [37,38]. (2) The intelligent analysis system transforms the data of animal response characteristics measured by cameras and microphones into key indicator information and analyzes them through AI and machine learning methods for the final decision of pig management [39]. (3) Optimize the production/reproduction process to avoid over-feeding, reduce farming waste and costs and make livestock farming more sustainable in economic, social and environmental aspects [17,40,41]. The AI, IoT and smart sensors continue to drive the development and application of precision pig farming systems. A vision of precision pig farming technology implementation can be seeb in Figure 2 [17,37,42].

## 3. AI-Based Intelligent Equipment for Precision Pig Farming

AI-based intelligent equipment occupies an important position in the precision pig farming system and is the material prerequisite for the realization of farming applications [17,37,42]. In the modern pig farming industry, equipment is a channel for obtaining and transferring data, and intelligent equipment with edge AI can process and analyze data on-site, which is helpful for making fast management decisions. The intelligent device has an effective execution function and can issue corresponding execution instructions to itself or other intelligent devices with the current monitoring conclusion so that the breeding environment can reach the best state of scientific breeding and thus reduce epidemic and increase income.

AI-based intelligent equipment for precision pig farming is currently implemented in product traceability, behavior monitoring and sound monitoring [35,43,44,45]. In terms of traceability, various processes such as production, processing, storage, distribution and retail have been improved by capturing information using IoT and various sensors (radio frequency identification (RFID)) [35]. When people find that food has quality or safety problems, they can locate the problem according to the product’s traceability system and then locate the cause.

Studies have shown that existing precision pig farming monitoring systems use a variety of sensor technologies to monitor multiple aspects of a pig’s life [27]. Among them, the ground weighing scales collect the weight of the pig, the microphone is used to monitor the pig’s sound, the thermal camera monitors the pig’s body temperature distribution and the infrared thermometer measure the pig’s temperature [11]. Moreover, each pig could be accurately and effectively monitored through RFID technology, and the temperature of the pig ear roots could be obtained by using the DS1992 iButton temperature sensor [46]. To study the relationship between feed intake and pig growth rate, RFID technology was used to identify pigs, load cells and feed through load cells collecting weight data and pig feed intake data, and transmitted the collected data to the server through the network [47]. To solve the problems of low automation and high cost of artificial pig feeding, researchers have designed an intelligent automatic feeding system for live pigs based on embedded advanced devices and RFID [45], RN30, RN31, RN32, RN33. The system is composed of a control system, sensors and other mechanical equipment. The control system is used for precise batching, and the mechanical equipment is used to realize automatic mixing and precise feeding.

**Figure 3:**
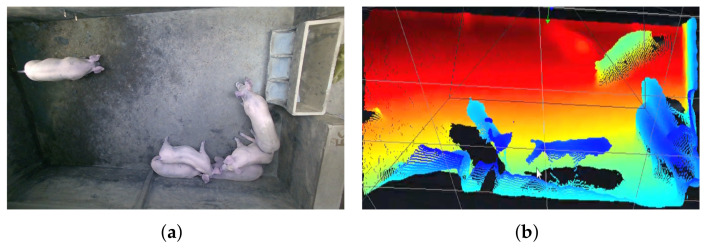
Camera top-view image and its corresponding 3D point cloud. (**a**) RGB image; (**b**) 3D point cloud.

With the development of visual AI, the use of pig sounds and images combined with deep learning models are more favorable in precision pig farming. There are two main ways to collect data using surveillance cameras. The first way is to fix surveillance cameras on top of the pigsty, which uses the top-view method to cover the entire pigsty (Figure 3a) and collect more information on pig backs [48,49,50,51,52]. The second approach collects pig videos from the side-view, which records more information on pig legs and trucks [53,54,55]. Similarly, the sound acquisition equipment is used to obtain the sound of pigs, and the characteristic images of pig sounds are extracted by mel frequency cepstral coefficients and other methods [56,57,58,59,60,61,62,63], which are combined with vision-based AI algorithms to realize the monitoring of pig sounds [56,57,59,62,64].

For pig behavior monitoring, the eYeNamic system is used both in pigs and poultry farming to monitor pig behavior [39]. The system acquires image data of pigs through cameras and uses analysis software to convert the acquired images into indicators for measuring animal position, movement and behavior. It can monitor pigs in real time to detect abnormal pig behavior and take corresponding rescue measures to reduce farming economic loss. Chen et al. deployed a novel spatial-aware temporal response filtering model to a counting robot with a monocular fisheye camera and monitored the pig’s health and safety through real-time counting and analysis of the pig number in the video [65]. In addition, GlassUp F4 smart glasses for augmented reality have the advantages of clear and fast data readability and data abundance in remote assistance to support field farmers [66]. Overall, the PLF system mainly includes data collection and processing, information analysis and decision-making and visualization display.

In terms of sound monitoring, the porcine cough monitor continuously and automatically measures pig respiratory health through sound analysis [43,67]. The SOMO^®^ respiratory distress monitor, developed by SoundTalks NV, automatically and continuously calculates the Respiratory Distress Index and provides an alert when there is a breathing problem in the barn [68]. In 2020, to monitor the respiratory diseases of pigs, the sound was collected by a MAX4466 electret microphone and a LIQI LM 320E Cardioid electret microphone [60,69]. The research showed that the combination of AI technology and sound collection equipment can realize the recognition of pig coughing sounds and effectively provide technical guidance for pig breeding.

In addition, the researchers studied the relationship between images of sick pigs and heart rate and respiratory rate associated with respiratory disease or other diseases [70,71]. In 2020, Jorquera-Chavez et al. studied computer-based techniques to measure changes in temperature, heart rate and respiration rate in pigs from thermal infrared and conventional images [70]. The study showed that computer vision techniques can provide important and usable data about physiological changes that may help in disease management. In 2021, Jorquera-Chavez et al. constructed and evaluated the usefulness of a system constructed based on techniques such as RGB (red, green and blue) and thermal imaging cameras, computerized tracking techniques and the photoplethysmography principle for remote monitoring of heart rate and respiration rate in pigs [71]. The experiment showed significant differences between sick and healthy pigs, with significant changes in their respiratory rate mainly released in the later stages of the disease; however, the technique still needs further study.

In pig weight prediction, 3D cameras are more beneficial than 2D cameras for estimating pig weight due to the extra depth information [72]. Kongsro et al. obtained the depth map and point cloud map (Figure 3b) of pigs using a Kinect 3D camera, which was then combined with machine learning technology to improve pig weight estimation accuracy [73]. Experiments have shown that the error of this method is estimated to be 4–5% of the average weight.

## 4. AI-Based Vision for Pig Detection and Tracking

**Figure 4:**
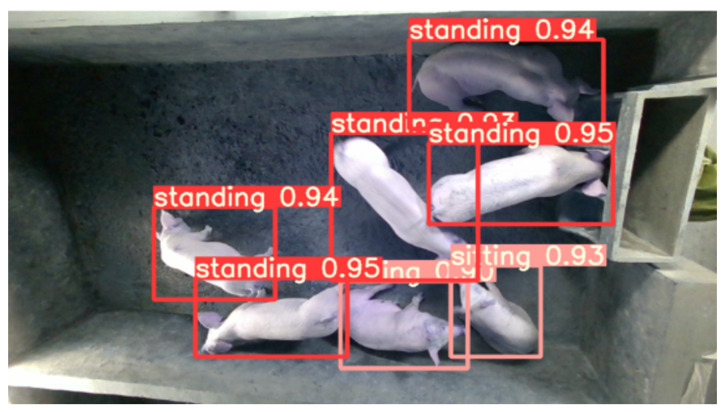
Examples of pig postures’ detection results.

Vision-based detection and tracking, as a non-contact approach, is a prerequisite for continuous access to information about pig behavior and welfare [74]. In pig monitoring applications, individual pig detection and tracking are the keys to moving away from group therapy to individual pig care and activity measurement [75,76]. The detection and tracking of all major parts of the pig’s body can help analyze the pig’s behavior [75,77]. AI-based vision makes automatic real-time detection (Figure 4) and tracking of pigs possible, which could monitor changes in individual pigs’ behavioral activity over time and use this as an indicator for health and welfare [78,79]. In addition, AI-based vision aids in the early detection of pig diseases, which leads to earlier, more effective interventions and reduces mortality [75]. Table 1 shows some works of AI-based pig detection and tracking based on visual data.

### 4.1. Pig Detection

Pig detection is the premise of tracking pig activities, monitoring pig behavior and continuously obtaining information [74]. Indeed, the accuracy of pig detection will directly affect the performance of animal tracking and behavior recognition [80]. In traditional machine learning, researchers used a combination of image differences with a median background and a Laplacian operator, pseudo-wavelet coefficients, ellipse fitting algorithm, Gaussian mixture model and other machine learning methods to detect and locate pig position [50,81,82,83,84]. However, these methods lack robustness in the complex farming scenario.

With the development of AI, especially the progress of convolutional neural network (CNN), varieties of AI-based approaches have been proposed for pig detection [75]. YOLOv3 and Faster R-CNN are two popular CNN models that have been used to detect pig position and demonstrated good detection performance [75,76,85,86]. Another method—The single shot multibox detector (SSD) algorithm, not only ensures pig detection accuracy but also accelerates the detection speed. Zhang et al. used the SSD and residual network (ResNet) to detect online multi-pig detection under sunlight and infrared (night) lighting conditions [74]. The results showed that the method achieved 94.72% detection accuracy.

More recently, in order to further improve pig detect location performance and facilitate tracking, researchers have proposed new methods to collect data and construct detection features. Sha et al. used a phase-sensitive Optical Time-Domain Reflectometer to collect real field vibration data and further processed the data into a spatiotemporal map to feed into the YOLOv3 network for pig detection [87]. This experiment initially validated the effectiveness of combining a Fiber Optic Distributed Vibration Sensor with an object detection scheme. In addition, Kim et al. proposed a more lightweight LightYOLOv4 model based on TinyYOLOv4 and pruning techniques (filter clustering), achieving 99.44% detection accuracy and a real-time detection speed of 30 frames per second on an embedded circuit board [88].

Since pigs are herd animals, there is a lot of occlusion and overlapping noise in the image data with captive pigs as the acquisition object [65,89]. In 2020, Chen et al. proposed a keypoint detection method based on a deep convolutional encoder–decoder network [65]. The approach determines the keypoints of each pig by keypoint heat map and offset vector field. This experiment showed that the keypoint-based detection method solved the occlusion and overlap problems more effectively than the bounding box-based detection method and achieved 84.7% mAP in the pig detection task. In 2021, Huang et al. evaluated the detection performance of Faster R-CNN and YOLOv4 models in object detection tasks [89]. The performance of Faster R-CNN and YOLOv4 models was 87% and 86% for mAP, respectively, in datasets with less occlusion noise. The performance of Faster R-CNN and YOLOv4 models decreased by 11.6% and 9.5%, respectively, in datasets with more occlusion noise. The experiments show that the Faster R-CNN is more affected by the occlusion compared with YOLOv4. In the same year, Hanse et al. used the YOLOv4 model trained into two models based on the original data and the enhanced data with reduced overexposure effects, respectively, and integrated the two models to improve their detection accuracy from 79.93–94.33% [90]. However, this integration method increases the execution method and model size. In 2022, to avoid image noise caused by impurities such as insect secretions, Zhao et al. proposed a noise image preprocessing method based on U-net and generative adversarial network (GAN) models to improve the accuracy of noise image detection [91]. The experiment showed that the method was able to improve the average detection accuracy of pigs from 76.6–90.6%.

### 4.2. Pig Tracking

The pig tracking algorithm can measure individual activity, which was negatively correlated with environmental factors such as temperature, relative humidity and ammonia [76,101]. In traditional image analysis methods, color features and contour features were used to capture all kinds of motion information of pigs [102]. Gao et al. segmented the pig’s head and tail, and used Hough clustering and roundness recognition algorithms to locate the trajectory of each pig [103]. Although this method could effectively segment adherent pigs, it still needs to be further optimized to improve the tracking accuracy.

In recent years, AI has achieved great success in visual tracking, including navigation, robotics, traffic control, etc. [104]. A variety of AI-based vision approaches have been proposed to realize real-time pig tracking and monitoring. The obtained movement trajectory and time of pigs could reflect each pig’s health status, increase the pig’s welfare and reduce the farming economic losses [65,99]. The main factors affecting object tracking are data processing, feature selection and detection model [80,105,106].

The consistency, integrity and quality of data are crucial to improving the tracking performance of AI models [106]. However, the collected data often has the problems such as occlusion and motion blur caused by rapid object movement [107]. In order to reduce the false positives caused by pig or camera movement or tracking failures, Chen et al. encoded each detected pig in the previous and subsequent frames uniquely associated the key points of the two frames with the energy maximization method based on bipartite graph matching and predicted the number of pigs with the novel spatial awareness temporary response filtering method [65]. Experiments showed that this method significantly avoided the false alarm of pig counts due to tracking failure. Liu et al. established a high-dimensional spatiotemporal feature model based on kernel principal component analysis and established an abnormal trajectory correction model from the five dimensions of semantics, space, angle, time and speed to avoid trajectory loss and drift [97]. The method mainly achieves optimal clustering of nonlinear trajectories by building a high-dimensional spatiotemporal feature model to enhance the accuracy of model tracking. Experiments showed that the method achieved 96.88% of trajectory tracking accuracy.

On the other hand, researchers studied the extraction of different features to improve the accuracy of tracking algorithms [105]. From the perspective of template matching, Chen et al. used 15 key points to divide a single pig into 10 parts, generated a series of prototype templates and selected an oriented fast and rotated brief algorithm to extract and describe key points of pig body parts and Hamming distance algorithm to match feature points, realizing real-time pig detection and tracking [77]. This experiment provides a new reference for keypoint-based detection and tracking. Wutke et al. used the Kalman filter algorithm to estimate the position of the pig shoulder key point in the current and previous frame. Thus the movement trajectory of the pig in a period of time could be tracked [95]. Experiments showed that this method achieved a MOTA score of 94.40%. Gan et al. formed a central feature vector from the central location of piglets and fed it into a CNN-based affinity estimation network to obtain the affinity prediction matrix while using the Hungarian algorithm to optimize the affinity prediction matrix, as well as designing a distance-based tracking state adjustment strategy to correct erroneous state predictions [86]. Experiments showed that the multiple object tracking accuracy (MOTA) of this method was 97.04%, the inference frame rate was 6.89 fps and its tracking performance and inference speed were better than the SORT model [108] and other methods.

In addition, the accuracy of the pig detection algorithm also has a direct impact on the accuracy of pig tracking [80]. In 2020, Zhang et al. constructed the object inverse probability projection map, then multiplied the Inverse Projection probability value of the pixel by the addition of surrounding pixels to obtain the object projection gray image to locate and track the pig [99]. Experiments showed that the average overlap rate of this method for tracking objects is 91.00%. Liu et al. simplified the pig tracking task to a detection problem for every two frames, which used the minimum tracking unit achieved 92.71% pig localization accuracy [100]. In terms of multi-object tracking, MOTS networks have been successful, but there was a key problem when it is used in pig farming applications; that is, the predicted masks do not fit objects (pigs) well [85,98]. The reason for this is the low resolution of feature maps in the mask branch.

For the accuracy of tacking models, online real-time continuous object tracking plays a key role [75]. The hierarchical data association algorithm combined a CNN detector, and a correlation filter-based tracker achieved efficient tracking of each pig online [74]. Cowton et al. used a deep simple online real-time tracking based on distance and vision to improve the automation of the model and online real-time pig tracking [75]. The experiment achieved 92% MOTA and online real-time tracking of multiple objects. In 2021, using the detection results as the input data of the SORT algorithm, continuous object tracking could be achieved [76]. The experiment showed that the method had an average tracking time of 57.8 min in a good environment. In 2022, Chen et al. built a semi-supervised pipeline to track pig activity over time, which does not require any pre-labeled videos [109]. Experiments showed that the rapid deployment of this system provided a reliable and easy solution for pig behavior monitoring.

### 4.3. Summary

AI-based detection and tracking algorithms overcome the difficulties of data processing and complexity of data feature extraction faced by traditional machine learning. Some state-of-the-art networks such as Faster R-CNN, Mask R-CNN and YOLO have been widely used in pig detection and tracking, which have demonstrated favorable performance in pig farming applications because of their strong automatic feature extraction capability [76,85,94].

However, the detection and tracking algorithms still face many challenges in practical applications: (1) The existence of complex environments such as lighting changes and pig occlusion in the actual application scenarios will affect the accuracy of AI models’ judgments [65,97]; (2) Using consumer-grade cameras to capture fast-moving pigs may present problems such as track fragmentation and track drift, and complex tracks may produce distorted data about the pigs, causing current tracking algorithms to potentially lose detection capability [74].

## 5. AI-Based Vision Pig Behavior Recognition

Pig behavior is one of the important factors for diagnosing their productivity, health and welfare [31]. A good understanding of natural and abnormal behavior changes in pigs can improve pig welfare, such as housing and diet [110]. In today’s trend of industrialized intensive pig farming, real-time tracking of pig behavior and analysis of pig behavior are critical for pig welfare, labor impact and corporate competitive advantage [111].

**Figure 5:**
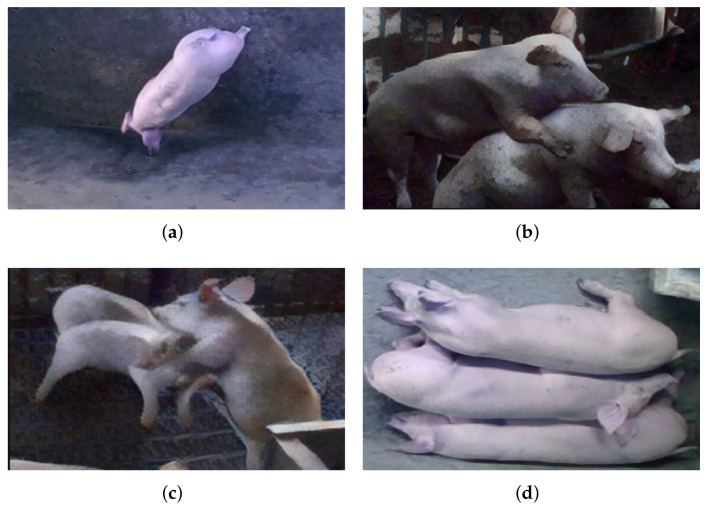
Examples of pig’s four different behaviors (i.e., drinking, mounting, aggressive and lying), (**a**) Pig drinking behavior; (**b**) Pig mounting behavior; (**c**) Pig aggressive behavior; (**d**) Pig lying behavior.

Pigs have different behaviors based on their living conditions. In most situations (normal environmental conditions), pigs perform daily behaviors, including drinking (Figure 5a), mounting (Figure 5b), aggression (Figure 5c) and laying (Figure 5d). These daily behaviors are important characteristics for judging a healthy diet and disease in pigs [112]. Table 2 shows the application statistics of AI vision-based pig behavior recognition. The following is a brief review of the different AI techniques used to identify pig behaviors.

### 5.1. Recognition of Pig Drinking Behavior

In the daily life of a pig, water is an essential part of the pig’s diet [113]. The amount of water consumed by each pig is not only related to pig feed intake but also could be used as a predictor of diseases [54,114]. To record a pig’s drinking behavior, the currently popular way is based on RFID tags (one sender placed on the pig’s ear and receivers installed next to the drinking fountains) [112]. Then, the correlation analysis between pig drinking behavior and their health could be established using a linear regression model [115,116,117,118]. However, RFID technology requires electronic tags punched into the livestock’s ears [119], and this invasive method of data collection can cause harm to the pigs [112].

In recent years, a camera combined with machine vision technology has been used to recognize pig drinking behavior. Kashiha et al. binarized the pig image and used the distance between the key points of the pig outline and the center of mass to judge the occurrence of pig drinking behavior [120]. In 2018, Yang et al. used the image occupation index to improve the accuracy of pig drinking behavior recognition [121]. In addition, Tan et al. used the similarity between two pig contour feature vectors to improve the recognition accuracy of pig drinking behavior [48]. However, these methods can not performe well in complex environments (e.g., illumination changing, occlusion) [48,120,121].

To improve the accuracy of pig drinking behavior recognition, Ji et al. used an image occupation index of the eating and drinking area and pig residence time in the area to judge the pig eating and drinking behaviors [54]. Chen et al. extracted spatial features based on the ResNet-50 model and used long short-term memory (LSTM) to identify pig drinking behavior [114]. The results showed that this method could detect pig drinking behavior under conditions of exposure and overlap.

### 5.2. Recognition of Pig Mounting and Estrus Behaviors

Pigs’ mounting behaviors often appear in overcrowded environments, and mounting behavior between pigs increases the risk of injury [136]. Moreover, improving the reproductive efficiency of sows can reduce management costs and feed costs per pig [137]. In natural mating, sows mate at least three times to increase the likelihood of fertilization [138]. However, the boar will produce behavior such as climbing and straddling when it is in estrus [139]. Therefore, accurate identification of mounting and estrus behaviors is essential to ensure sow fertilization and pig health and welfare [49].

In the early stage of pig mounting and estrus recognition, background subtraction and ellipse fitting techniques are often used to locate pigs in the image. The Euclidean distance between the head and the tail and the lengths of the major and minor axes of the fitted ellipse between the head and the side were used as the characteristics of mounting behavior, and pig mounting behavior was identified based on this [136].

For image-based automatic detection of pig mounting behavior, Li et al. used ResNet feature pyramid networks model to segment pig images, then realized the recognition of mounting behavior according to the mask pixel area [133]. At the same time, the mask region-convolutional neural network (Mask R-CNN) segmentation network and the kernel extreme learning machine were used to improve the accuracy of mounting behavior recognition [49]. This model could effectively solve the segmentation problem of pig occlusion. In terms of pig estrus behavior recognition, Zhuang et al. followed the obvious characteristics of pig ears during estrus, collected pig ear image data during estrus and non-estrus, and simplified the Alexnet network structure to improve the speed of pig estrus behavior recognition [126].

Compared with images, temporal sequence information in videos contains more detailed features, which could further enhance the recognition performance of pig mounting behavior [129]. For video data, spatiotemporal features are more effective for mounting behavior in video sequences than only extracting spatial features of images [139]. Wang et al. used the moth-flame optimization-based LSTM classification model to identify pig estrus behavior [125]. Experiments showed that this method could effectively identify the pig estrous behavior. In the same year, Li et al. proposed a spatiotemporal convolution behavior recognition model based on the SlowFast model, which achieved an accuracy of over 97%. The model was compared with the single stream 3D convolution network (SlowFast-50, R3D-18), and experiments showed that the accuracy and generalization ability of the model in pig mounting behavior recognition had been improved [129]. In addition, Yang et al. selected the distance, overlapping area and the intersection angle of two pigs in a single frame as spatial features, used the change rate of these features in adjacent frames as temporal features, then built a classifier based on XGBoost to recognize the mounting behavior in pig video [139]. Experimental results showed that the detection accuracy of pigs was 97%, and the average accuracy of pig mounting behavior detection was 95.15%.

### 5.3. Recognition of Aggressive Pig Behavior

Pigs are more likely to exhibit aggressive behavior in intensive closed farming [140]. Aggressive behavior in pigs can cause problems such as uneven food distribution, skin injury, wound infection, etc., resulting in reduced pig welfare and economic losses [141]. Therefore, there has been great interest in using computer vision and AI technology to recognize aggressive pig behavior [127].

Traditional feature extraction-based machine learning models have problems such as too few feature types, complex artificial feature selection and weak generalization ability [140,141,142,143,144]. In recent years, with the development of deep learning in the field of machine vision, the AI method effectively avoids the complex problems of feature selection and data processing in traditional machine learning, which significantly increases the accuracy and generalization ability with its strong feature learning ability [134].

In AI-based approaches, 2D or 3D convolution, multi-scale fusion and spatiotemporal feature extraction are used to extract advanced features of images so as to improve the recognition accuracy of aggressive behavior [122,127,134]. Gao et al. added multi-scale feature fusion, Dropout, and Batch Normalization to the convolutional 3D (C3D) network structure, achieving 95.70% accuracy for aggressive pig behavior recognition models in a complex environment [134]. Chen et al. proposed VGG-16 and LSTM-based approaches to directly extract the spatial features and feed these features into a LSTM model for extracting the spatiotemporal features, which obtained 98.4% accuracy for aggressive behavior recognition [127].

In addition, in order to achieve high-precision and complex social behavior recognition, Gan et al. used key points of a pig’s body to represent the motion decomposition of pigs and extracted high-quality spatiotemporal features (linear motion intensity, angular motion intensity and spatial affinity) using convolution networks and adaptive spatial affinity kernel functions to identify social behavior containing aggressive behavior [122]. Studies have shown that this method could effectively detect and identify behavior, such as aggression in pigs, providing key indicators for improving pig health and welfare. D’Eath et al. used 3D point cloud data and a series of linear mixed effect models to study the relationship between the posture of a pig’s tail and other factors, such as the pig’s aggressive behavior [123]. The experimental results showed that in commercial farming, the tail posture disturbance in normal time was related to tail biting and other adverse health/welfare signs, which formed the basis of a decision support system.

### 5.4. Recognition of Pig Nursing, Lying down and Other Behaviors

Productivity in commercial piglets’ production is largely dependent on the number of weaned piglets per sow. However, one of the main reasons for high pre-weaning mortality in piglets is starvation [145]. In addition, pig lying and other behaviors are also important characteristics in judging the healthy diet and pig disease [112]. In the early stage, Yuan et al. used the Zernike moment and support vector machine to recognize four poses of pigs [146]. However, facing complex pose and behavior recognition problems, traditional machine learning algorithms often cannot perform well. With the development of AI algorithms, a variety of deep learning-based approaches have been proposed to detect pig behavior to maximize the welfare and economic benefit of pigs [111]. The deep learning-based pig feeding behavior, posture and other behavior recognition often train models using images, video and 3D data [124,129,135].

Regarding image data-based approaches, Xue et al. proposed the ZF model with deeper layers and two residual learning frameworks to realize the effective recognition of the behavior of lactating sows, which achieved an average accuracy of the methods of 96.73%, 94.62%, 86.28%, 89.57% and 99.04% for five postures of lactating sows (e.g., standing, sitting, lying down, lying on the stomach and lying on the side, respectively) [135]. However, this model is large, and it is difficult to migrate and deploy into the embedded systems. Riekert et al. regarded the pig lying posture as an object detection problem that used NASNet and Faster R-CNN to achieve 80.20% mAP for the detection of pig position and postures [53].

From the perspective of video data, Li et al. proposed a spatiotemporal convolution network for pig multi-behavior recognition based on the slowfast-two path structure and 3D ResNet (R3D) model [129]. The experimental results showed that the model still had significant generalization ability in the subsequent pig detection tasks. Similarly, considering the motion information of pig behavior in the video data, a dual-stream convolutional network model based on deep learning was proposed to recognize pig behavior [130]. In the same year, Alameer et al. mapped the breast area to the optical flow frame corresponding to the lactation behavior and extracted the temporal characteristics of the sows’ exercise intensity and occupancy index to extract temporal and spatial characteristics for distinguishing breastfeeding and similar behavior [131]. In 2021, Gan et al. located the sows lactation area through the spatial positioning network composed of sow detectors and key point detectors, and used spatiotemporal feature information of sow’s to identify a sow lactation behavior classifier [124].

In terms of 3D data (e.g., RGB-D images), based on RGB-D images of the pigs, Zheng et al. used the improved Fast R-CNN architecture as a sow frame-level pose detector, which achieved 92.70% mAP for the detection of four postures (standing, sitting, ventral lying and lateral lying) [128]. Experimental results showed that this method could monitor pig postures in real time and provide effective reference information for farming.

### 5.5. Summary

Pig behavior is one of the important factors in productivity, health and welfare [31]. At present, AI algorithms apply deep learning models with images or videos for recognizing pig behaviors. In terms of images, researchers mainly applied Faster R-CNN, Mask R-CNN, YOLO, ResNet, VGG and other algorithms to identify pig behavior recognition [53,54,55,114,124,127,133]. For video-based approaches, LSTM, R3D, C3D, etc., are often used to extract spatial-temporal features, which often achieve better recognition performance than that of image-based approaches [114,125,127,129,132,134].

However, there are still many challenges in pig behavior recognition: (1) Recognition accuracy of similar and multi-behaviors in complex environments need to be further improved [122]; (2) How to solve the problem of crowding and confinement among pigs resulting in the loss of many key behavioral traits is a challenge [114,139]; (3) The high-speed movement of pigs causes deformation and drift of pig bodies, which increases the difficulty of behavior recognition [114].

## 6. AI-Based Sound for Pig Disease and Estrus Diagnosis

In the process of pig farming, in addition to vision for monitoring abnormal pig behaviors, sound is also the most obvious external manifestation for pig diseases [12]. In some pig farms, the pig mortality rate due to respiratory diseases is as high as 15.00%, which has caused huge farming economic losses [147,148]. Pig’s coughing sounds could be used as a feature to identify pig respiratory diseases [149,150,151]. For monitoring and recognition of pig coughs and estrus sounds, data preprocessing and sound recognition models are two important aspects.

**Figure 6:**
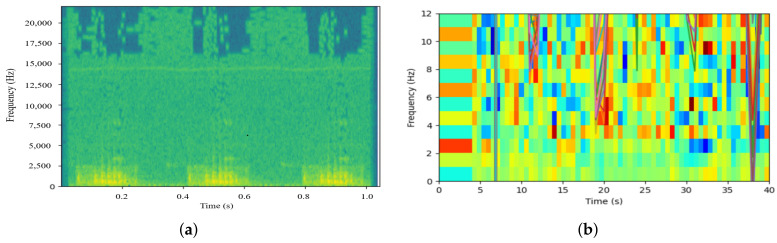
Spectrogram and mel frequency cepstral coefficients of pig cough sound. Hertz (Hz) is the unit of frequency. Second (s) is the unit of time. (**a**) Spectrogram of pig cough sound; (**b**) Pig cough sound mel frequency cepstral coefficients diagram.

In terms of data preprocessing, as the pig cough sound spectrum illustrated in (Figure 6a), the environmental noise frequency band of pig farming is usually below 5 KHz, which overlaps with the pig cough sound frequency band [57,58,59,152]. In order to better remove fan noises in pig farming, Dong et al. proposed a Discrete Cosine Transform-based enhancement algorithm for pig cough sound signal denoising [153]. Yan et al. used the log energy entropy quadratic wavelet packet denoising method to remove piglets and pink noise; meanwhile, filters and wavelet thresholds were used to remove mechanical running noise [154,155]. Ma et al. used 150 order FIR filter band-pass filtering to effectively remove the noise outside the frequency band of pig cough sounds [156]. Additionally, Butterworth band-pass filter and multi-window spectrum-based psychoacoustic speech enhancement algorithms are also used to remove the noise [56,57,58,59,152].

On the other hand, sound recognition models such as support vector machine model, decision tree, double threshold algorithm, dynamic time warping, the sparse representation classifier and fuzzy c-means clustering also play an important role in the recognition of pig cough sounds [157,158,159,160,161]. Here, some common sound features such as power [155], Multidimensional Short-Term Energy, mel frequency cepstral coefficients (Figure 6b) [56,57,58,59,60,61,62,63] and short-term zero-crossing rate [156] are often used for model training. However, the above methods lack robustness in the complex farming environment.

With the development of AI technology, in order to improve the monitoring of pig cough sounds in a complex environment, different deep learning models for data feature extraction and sound recognition have been proposed recently [63,69,152]. Table 3 shows the application statistics of AI algorithms in pig sound recognition.

Researchers combined the deep belief network model, backpropagation neural network, bidirectional long short-term memory model and deep neural network hidden Markov model to detect and recognize pig cough sounds [56,57,59,62,64]. Similarly, the sound feature image extracted based on the fast Fourier transform algorithm was input into the MobileNetV2 network model line, which could realize the recognition of various pig sounds well [152]. In addition, Chen et al. used the deep transfer learning and CNN model to recognize the estrous sound of pigs [63]. The results showed that the deep learning-based approach could distinguish sow estrus sounds and boar estrus sounds very well. In the same year, Yin et al. proposed an Alexnet-based model using spectrum features, which used the advantages of a convolutional neural network to improve the recognition accuracy of pig cough sound [69].

## 7. Challenges and Development Opportunities

This paper reviews the application of AI in precision pig farming. Although AI-based vision and sound have demonstrated good performance in pig detection and behavior recognition, there are still many challenges and opportunities for the systematic application of precision pig farming due to the size of the farming, the education level of the farming personnel, the standard of intelligent equipment and farming data.

### 7.1. Key Challenges in the Pig Monitoring System

The main challenges facing the pig industry are as follows.

- The reliability, ease of maintenance and use of intelligent devices are important challenges. In terms of reliability, the sensor or devices may need to be installed on the roof of the pig house or another unfriendly environment (e.g., high temperature, humidity, dust and unstable electricity), which will cause some erosion and damage to the hardware (such as the sensor) [34,162]. On the other hand, livestock farming is often located in remote rural areas, which causes inconvenience to personnel maintenance [34]. Meanwhile, the high-technology devices or systems need skilled farming staff to operate; how to develop precision pig devices or systems that are not limited by the level of education and ease of use for farmers is an important challenge [163].
- In terms of pig farming data, there are also problems such as a lack of extensive high-quality datasets and data standards. As the commercial or bio-security restrictions of pig farming increase, so do the difficulty of data collection and publication [164]. With the wide usage of 3D sensors, data storage or compressed standards are also demanded. Take the realsense D455 3D camera as an example; with 15 fps acquisition frequency and 848 × 480 image resolution, the storage capacity is about 50 G for one hour per day, and the hard disk capacity is about 1.2 T for one day. Therefore, data collection and data storage are important challenges in establishing a PLF system [165].
- Pig’s digital growth model is urgently demanded. The growth cycle of pigs usually lasts 5 or 8 months. Along with pigs’ body characteristics and weight changes, the nutritional requirements of pigs at different growth stages are different [166,167,168]. Therefore, a good pig growth model not only can monitor the weight change of pigs but could also guide the feeding nutrition management for the pig industry to achieve low-cost and sustainable farming [33].

### 7.2. Development Opportunities for the Pig Industry

With the development of related technologies, such as smart sensors and AI, precision pig farming continues to develop in the direction of knowledge-based, technology-based and modern smart livestock farming [169]. The main opportunities for the pig industry development are as follows.

- Intelligent sensing technology, IoT and AI technology are integrated with different sensor modes and expert knowledge [35,170] to develop towards standardization, larger scale and intelligence [171].
- Continue to develop automatic recognition approaches for pig’s external appearance phenotype and inner physiology status in the complex farming environment [10,31]; Establish multi-modal methods that could utilize vision and sound signals to detect behavior and diagnosis diseases at different growth stages, further quantify the identification results [25,172].
- Improve the automatic pig farming level: The automation of machinery and real-time monitoring devices can be further developed to reduce labor requirements [37]. In addition, a digital pig growth model, and health and welfare evaluation system should be established to increase product traceability and promote automatic pig management levels [55,132].

## 8. Conclusions

The paper systematically summarizes the current research progress of the main sensor devices used in pig farming and the AI-based vision and sound for detection and tracking, behavior, etc. In modern farming, temperature sensors, weight sensors and RFID sensors are used to obtain data about the pigsty environment or pig, and 2D and 3D cameras are used to obtain data about the pigs. On this basis, combined with AI techniques, the deep learning algorithms for pig detection and tracking, pig drinking, mounting, aggression, lactation, lying down and other behaviors are expounded. In addition, the deep learning algorithm for recognizing respiratory diseases through pig sound is described. Much has been achieved so far in the real-time monitoring of pigs, but further improvements to the practicality and stability of the farming systems for automatic and sustainable pig industry development are needed. 

## Figures and Tables

**Table 1:** Main research work on pig image detection and tracking.

Authors, Year	Dataset Size (Images Number)	Method	Breed	Result
Zhao et al., 2022 [91]	18,000	Mask R-CNN and GAN	-	Average Precision = 90.6%
Lei et al., 2022 [92]	416,873	U-Net and UNet-Attention	Yorkshire pig	Average Precision = 94.80%
Ocepek et al., 2022 [93]	583	Mask R-CNN and YOLOv4	Crossbred Norsvin Land-race × York-shire sow in-seminated with Duroc boar semen	Precision = 96.00%
Ding et al., 2022 [94]	5000	YOLOv5 and FD-CNN	Pregnant Large White sow	Precision = 93.60%
Wutke et al., 2021 [95]	12,285	CNN and KF	-	MOTA = 94.40%
Sun and Li., 2021 [96]	-	A multi-object tracking algorithm, which based on joint probability data association and particle	-	Correct tracking rate = 99.00%
Van Der Zande et al., 2021 [76]	4000	YOLOv3 and SORT	Crossbred pig	mAP = 99.70%
Sha et al., 2021 [87]	5988	YOLOv3	-	-
Liu et al., 2021 [97]	5000	ResNet-50 and DLC-KPCA	Weaned Yorkshire piglets	Accuracy = 96.88%
Jung et al., 2021 [85]	2182	Faster R-CNN and OCTA	-	Accuracy = 77.00%
He et al., 2021 [98]	1400	Mask R-CNN and Track R-CNN	-	MOTSA = 94.90%
Gan et al., 2021 [86]	100 video clips	Faster R-CNN and OPTN	Meihua sow	MOTA = 97.04%
Zhang et al., 2020 [99]	425 GB	CamTracor-PG	-	The average overlap rate = 91.00%
Liu et al., 2020 [100]	320	SSD + ResNet-50 and MTU	(Landrace × Large White) × Piétrain crossbreds	Precision = 96.38%
Chen et al., 2020 [65]	51 video clips	Bottom-up keypoints detection CNN architecture and STRF	-	mAP = 84.30%
Chen et al., 2020 [77]	15,000	YOLACT	Landrace × Yorshire crossbred pig	Accuracy = 90.00%
Zhang et al., 2019 [74]	18,000	SSD and Correlation Filter	Large White × Landrace breed	Precision = 94.72%
Cowton et al., 2019 [75]	3292	Faster R-CNN, SORT and Deep SORT	-	mAP = 90.10%

Notes: GAN means generative adversarial network; FD-CNN means frame differences in combination with convolutional neural network; KF means kKalman filter algorithm; SORT means simple online real-time tracking; CNN means convolutional neural networks; ResNet means residual nets; DLC-KPCA means deepLabcut-kernel principal component analysis; OCTA means object center-point tracking algorithm; R-CNN means regionconvolutional neural network; Mask R-CNN means mask region-convolutional neural network; OPTN means online piglet tracking network; CamTracor-PG means camshift tracking approach based on correlation probability graph; SSD means single shot multibox detector; MTU means minimum tracking unit; STRF means spatialaware temporal response filtering; mAP means mean average precision; MOTSA means multi-object tracking and segmentation accuracy; MOTA means multi-object tracking accuracy; YOLACT means you only look at coefficients; Deep SORT means deep simple online real-time tracking; - means that the authors did not state specific data or did not mention this property in the text.

**Table 2:** The main research work of pig behavior recognition.

Authors, Year	Data Type	Behavior	Method	Breed	Accuracy
Riekert et al., 2021 [55]	2D	Lying	Faster R-CNN, NASNet	Pig (GermanHybrid × German Piétrain)	84.00%
Gan et al., 2021 [122]	2D	Snout-snout and snout-body social nosing, snout-snout and snout-body aggressive/ playing behavior	ResNet-101	Meihua sow	93.09%
D’Eath et al., 2021 [123]	3D	Scratched tails	Linear mixed models	Grower/finisher pig	-
Gan et al., 2021 [124]	3D	Nursing	ResNet-50, FlowNet2.0	Meihua sow	97.63%
Ji et al., 2020 [54]	2D	Eating and drinking	YOLOv2	Yorkshi sow	94.59%
Chen et al., 2020 [114]	2D	Drinking	ResNet-50 + LSTM	Mixed nursery pig	92.50%
Wang et al., 2020 [125]	2D	Estrus	MFO-LSTM	Landrace pig	98.02%
Zhuang et al., 2020 [126]	3D	Estrus	AlexNet	Large white sow	93.33%
Chen et al., 2020 [127]	2D	Aggressive	VGG16 + LSTM	Mixed nursery pig	98.40%
Zheng et al., 2020 [128]	3D	Walking, keep standing, keep sitting, keep ventral recumbency behavior et al.	Fast R-CNN and HMM	Small-ears spotted pig	92.70%
Riekert et al., 2020 [53]	2D	Lying	Faster R-CNN + NAS	Fattening pig	80.20%
Li et al., 2020 [129]	3D	Feeding, lying, motoring, scratching and mounting behavior	PMB-SCN	Fragrance pig	97.63%
Zhang et al., 2020 [130]	3D	Feeding, lying, walking, scratching and mounting behavior	TSCNM	Fragrance pig	98.99%
Alameer et al., 2020 [131]	2D	Nursing	SVM	Sow pig	96.40%
Chen et al., 2020 [132]	2D	Feeding	Xception + LSTM	Mixed nursery pig	98.40%
Li et al., 2019 [49]	2D	Mounting	Mask R-CNN and KELM	Minipigs pig	91.47%
Li et al., 2019 [133]	2D	Mounting	Mask R-CNN and ResNet-FPN	-	94.50%
gao et al., 2019 [134]	3D	Aggressive	3D CONVNet	-	96.78%
Tan et al., 2018 [48]	2D	Drinking	Douglas-Peukcer	-	93.75%
Yang et al., 2018 [121]	2D	Drinking	Google Lenet	-	92.11%
Xue et al., 2018 [135]	3D	Standing, sitting, prone and side lying behavior	Faster R-CNN, ZF-D2R	Sow	96.73%

Notes: NAS means neural architecture search; ResNet means residual nets; LSTM means long short-term memory; MFO means moth-flame optimization; HMMmeans hidden Markov model; PMB-SCN means a SlowFast networkbased spatiotemporal convolutional network for the pig’s multi-behavior recognition; Xception means Extreme version of Inception; KELM means kernel extreme learning machine; NAS means neural architecture search; TSCNM means two-stream convolutional network models; SVM means support vector machine; Mask R-CNN means mask region-convolutional neural network; ResNet-FPN means residual net feature pyramid networks; ZF-D2R means ZF model with deeper layers and two residual learning frameworks; keep standing means maintaining a standing position continuously for a certain period of time without making any other movements; keep sitting means maintaining a sitting position continuously for a certain period of time without making any other movements; keep ventral recumbency means maintaining a ventral recumbency position continuously for a certain period of time without making any other movements; - means that the authors did not state specific data or did not mention this property in the text.

**Table 3:** The main research work of pig sound recognition.

Authors, Year	Sound Category	Method	Breed	Result
Yin et al., 2021 [69]	Cough	AlexNet	-	Accuracy = 95.40%
Chen et al., 2021 [63]	Estrus sound	VGG16, DTL-CNN	Sow	Accuracy = 96.62%
Zhao et al., 2020 [62]	Cough	DNN-HMM	Landrace pig	Average WER = 8.03%
Shen et al., 2020 [61]	Cough	MFCC-CNN	-	Accuracy = 97.72%
Hong et al., 2020 [64]	Cough, grunt, scream	MnasNet	Pig (Yorkshire, Landrace, and Duroc)	Accuracy = 94.70%
Li et al., 2020 [60]	Cough	SVDD	-	Accuracy = 93.70%
Cang et al., 2020 [152]	Cough, sneeze, hunger, choking, and screams	MobileNetV2	Three-way sow	Accuracy = 97.30%
Zhang et al., 2019 [59]	Cough, sneeze, hunger, choking, and screams	SVDD, BPNN	-	Accuracy = 95.40%
Wang et al., 2019 [58]	Cough	PCA, SVM	Landrace weaners	Accuracy = 95.00%
Li et al., 2019 [57]	Cough	BLSTM-CTC	Landrace	Accuracy = 93.77%
Cordeiro et al., 2018 [161]	Pig vocalization	decision-tree	Sow	Accuracy = 81.92%
Li et al., 2018 [56]	Cough	PCA, DBN	Landrace	Accuracy = 94.29%
Dong et al., 2017 [153]	Cough, wind noise	DCT	-	-
Hui et al., 2016 [156]	Cough	-	-	Accuracy = 96.00%
Yan et al., 2016 [155]	Nursing grunt, drinking, feeding and sham chewing	The sub-band clustering method based on skewness and SVM	-	Accuracy = 95.17%

Notes: DTL-CNN means deep transfer learning and convolutional neural network; DNN-HMM means deep neural network hidden Markov model; MFCC means mel frequency cepstral coefficient; MnasNet means Neural Architecture Search for Mobile; SVDD means support vector data description; MobileNetV2 means mobile networks; BPNN means back-propagation neural network; PCA means principal component analysis; SVM means support vector machine; BLSTM-CTC means birectional long short-term memory-connectionist temporal classification; DBN means deep nelief network; DCT means discrete cosine transform; WER means word error rate; - means that the authors did not state specific data or did not mention this property in the text.

## Data Availability

Not applicable.
